# A Rare Case of Cyclophosphamide-Induced Posterior Reversible Encephalopathy Syndrome in a Patient With Acute Lupus Nephritis Flare

**DOI:** 10.7759/cureus.34372

**Published:** 2023-01-30

**Authors:** Mpey K Tabot Tabot, Priscilla A Ababio, Shervonne Waldron, Lamiaa Rougui, Alem Mehari

**Affiliations:** 1 Internal Medicine, Howard University Hospital, Washington, D.C., USA; 2 Pulmonary Medicine, Howard University Hospital, Washington, D.C., USA; 3 Pulmonary/Critical Care, Howard University Hospital, Washington, D.C., USA

**Keywords:** lupus nephritis flare, posterior reversible encephalopathy syndrome, lupus nephritis, prednisone, endothelial dysfunction, cyclophosphamide, posterior reversible encephalopathy syndrome (pres)

## Abstract

Posterior reversible encephalopathy syndrome (PRES) is a syndrome encompassing both clinical and radiological manifestations with white matter vasogenic edema predominantly of the posterior and parietal lobes of the brain. It may accompany several medical conditions including immunosuppressive/cytotoxic drugs. We present a case of cyclophosphamide-induced PRES in a patient treated for acute lupus flare with biopsy-proven lupus nephritis.

A 23-year-old African American female presented with non-specific symptoms over a six-month period on a medical background of systemic lupus erythematosus and biopsy-proven focal lupus nephritis class III on hydroxychloroquine, prednisone, and mycophenolate mofetil for which she was non-compliant. She was borderline hypertensive, tachycardic, saturating well on ambient air, and alert and oriented. Laboratory workup revealed electrolyte imbalance, elevated serum urea, creatinine, and B-type natriuretic peptide, low serum complements, and elevated double-stranded DNA (dsDNA) with negative lupus anticoagulant, anti-cardiolipin, and B2 glycoprotein antibody. Chest imaging revealed cardiomegaly with small pericardial effusion, left pleural effusion, and trace atelectasis, with no deep vein thrombosis on Doppler ultrasound. She was admitted to the intensive care unit for lupus flare with severe hyponatremia and was continued on mycophenolate mofetil, hydroxychloroquine, and prednisone 60 mg for induction therapy as well as intravenous fluids. Hyponatremia resolved, and blood pressure was controlled. She became fluid overloaded and anuric, with pulmonary edema and worsening hypoxic respiratory failure not responding to diuretic challenges. Daily hemodialysis was started, and she was intubated. Prednisone was tapered down, mycophenolate was switched to cyclophosphamide/mesna. She became agitated, restless, and confused, with waxing and waning consciousness and hallucinations. She was continued on bi-weekly cyclophosphamide for induction therapy. After the second dose of cyclophosphamide, her mentation worsened. Non-contrast MRI showed extensive bilateral cerebral and cerebella deep white matter high-intensity signals suggestive of PRES, which was new compared to one year prior. Cyclophosphamide was held and her mentation improved. She was successfully extubated and discharged to a rehabilitation center.

The exact pathophysiological mechanism of PRES is not known. Endothelial damage and vasogenic edema have been hypothesized as possible mechanisms. Severe anemia, fluid overload, and renal failure are some of the causes of endothelial dysfunction and vasogenic edema with disruption of the blood-brain barrier, which were found in our patient, but repeated dosing of cyclophosphamide worsened her condition. Discontinuation of cyclophosphamide led to a significant improvement and complete reversal of her neurologic symptoms, implying that prompt recognition and management of PRES is vital to prevent permanent damage and even death in these patients.

## Introduction

Posterior reversible encephalopathy syndrome (PRES) is a radio-clinical syndrome that presents with varying degrees of neurological signs and symptoms and an imaging finding of vasogenic white matter edema that is predominant in the posterior lobes of the brain [1] but also in the anterior aspects of the brain, making it a misnomer [2]. Intravenous cyclophosphamide has been featured in the literature as an inciting event for PRES in patients with and without systemic lupus erythematosus (SLE). We present a case of acute severe biopsy-proven lupus nephritis flare in a patient who developed PRES after receiving cyclophosphamide for induction therapy.

## Case presentation

A 23-year-old African American female presented with non-specific gastrointestinal symptoms, including vomiting and diarrhea with occasional hematochezia. She also had a productive cough with an occasional small amount of hemoptysis, chills, and generalized weakness that progressively worsened over a period of six months. There was no history of seizures or psychiatric problems. She had a medical background of SLE and biopsy-proven lupus nephritis with mesangial glomerulonephritis diagnosed at the age of 14 and was placed on hydroxychloroquine, prednisone, and mycophenolate mofetil. A repeat kidney biopsy two years later showed focal lupus nephritis class III with focal segmental glomerulosclerosis. The patient had stopped using her medications five months prior to presentation due to side effects of fatigue, insomnia, and headaches. She was restarted on steroids due to persistently high inflammatory markers and proteinuria.

On admission, she was borderline hypertensive, tachycardic, and saturated well on ambient air. She was alert and oriented, her lungs were clear, and her heart was regular and tachycardic. Laboratory workup revealed severe hyponatremia, hyperkalemia, hyperphosphatemia, elevated serum urea, creatinine, D-dimer, double-stranded DNA (dsDNA), and B-type natriuretic peptide, with low serum complements, as shown in Table 1. Lupus anticoagulant, anti-cardiolipin, and B2 glycoprotein antibody were negative. Chest imaging revealed cardiomegaly with small pericardial effusion, left pleural effusion, and trace atelectasis, with no deep vein thrombosis in bilateral lower extremities on Doppler ultrasound.

**Table 1:** Patients' laboratory values and reference ranges. dsDNA: double-stranded DNA.

Laboratory tests	Patients’ values	Normal values
B-type natriuretic peptide	1364 Pg/mL	<100 Pg/mL
C3	18.53 mg/dL	79-152 mg/dL
C4	<8 mg/dL	16-38 mg/dL
dsDNA	947 IU/mL	<4 IU/mL
D-dimer	6.76 microgram/mL	0.0-0.48 microgram/mL
Serum sodium	115 mEq/L	135-145 mEq/L
Serum potassium	5.2 mEq/L	3.5-5.1 mEq/L
Serum phosphorus	7.0 mg/dL	2.5-4.5 mg/dL
Blood urea nitrogen	48 mg/dL	7-25 mg/dL
Serum creatinine	3.76 mg/dL	0.6-1.1 mg/dL

**Figure 1:**
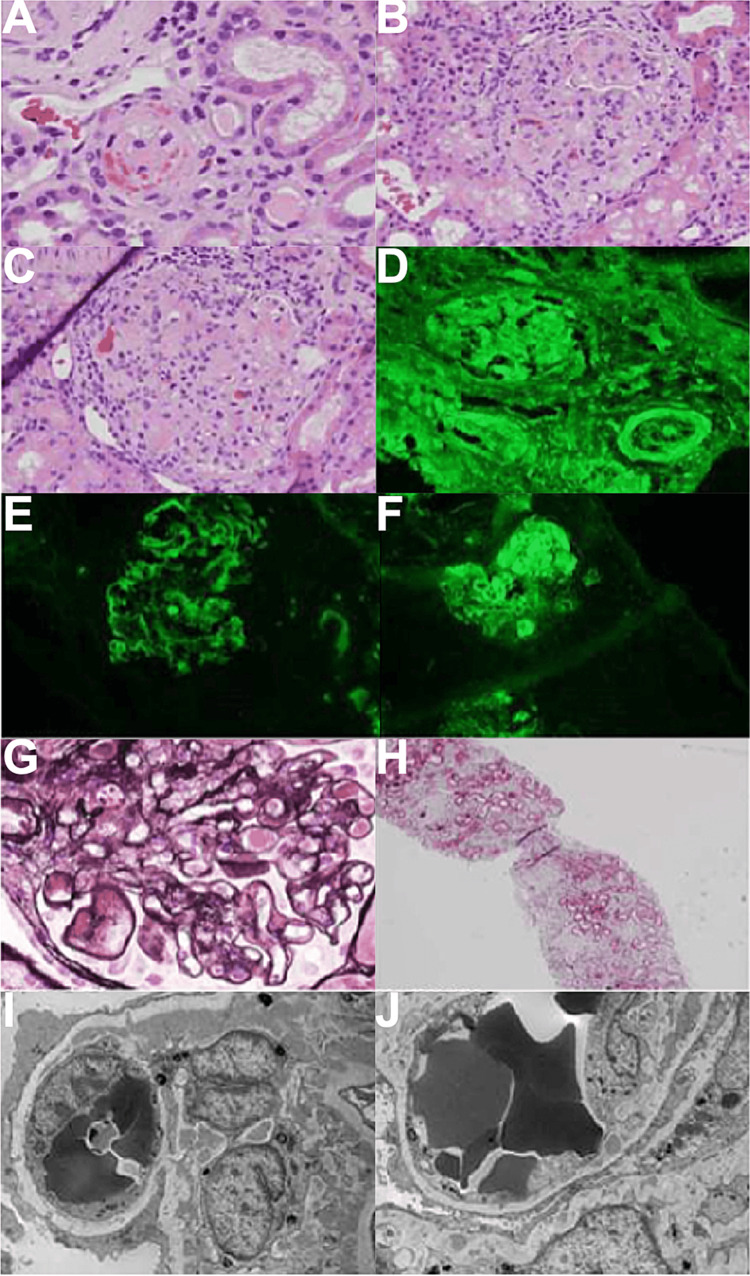
Renal biopsy showing diffuse proliferative lupus nephritis International Society of Nephrology/Renal Pathology Society (ISN/RPS) class IV with 58% global glomerulosclerosis, moderate interstitial fibrosis/tubular atrophy involving 45% of the renal cortex, and acute thrombotic microangiopathy. A: H&E stain, 400x original magnification - thrombotic microangiopathy with thrombus and fragmented red blood cells. B: H&E stain, 400x original magnification - necrotizing glomerulonephritis. C: H&E stain, 400x original magnification - fibrinoid necrosis of glomerular tuft. D: IF stain, 400x original magnification - IgG. E: IF stain, 400x original magnification - IgA. F: IF stain, 400x magnification - C1q. G: Silver stain, 400x original magnification - double contours. H: Trichrome stain, 400x original magnification - interstitial fibrosis. I: TEM stain, 400x original magnification - subendothelial and mesangial electron-dense deposits. J: TEM stain, 400x original magnification - subendothelial and intramembranous electron-dense deposits. H&E: hematoxylin and eosin; IF: immunofluorescence; TEM: transmission electron microscopy.

She was admitted to the intensive care unit for lupus flare with severe hyponatremia and placed on high-dose intravascular (IV) steroids for three days and bolus fluids of normal saline. Hyponatremia resolved and she was sent to the floor. Her blood pressure remained elevated ranging from 140s to 170s systolic and 90s to lower 100s diastolic in mmHg. A repeat kidney biopsy was done, which revealed diffuse proliferative lupus nephritis International Society of Nephrology/Renal Pathology Society (ISN/RPS) class IV with 58% global glomerulosclerosis, moderate interstitial fibrosis/tubular atrophy involving 45% of renal cortex and acute thrombotic microangiopathy, index of activity 12/24, and index of chronicity 9/12, as shown in Figure 1. The patient was continued on mycophenolate mofetil, hydroxychloroquine, and prednisone 60 mg for induction therapy and plasmapheresis for thrombotic microangiopathy complicating lupus nephritis.

Two weeks into admission, the patient became fluid overloaded, and urine output continued to drop until she became oliguric and eventually anuric with pulmonary edema on imaging not responding to the diuretic challenge with worsening hypoxic respiratory failure. She was started on daily hemodialysis and was transferred back to the medical intensive care unit where she was intubated for worsening respiratory distress and was placed on a heparin drip while awaiting pulmonary embolism (PE) ruled out. Platelets began to drop significantly, and heparin was switched to argatroban drip while heparin-induced thrombocytopenia (HIT) workup was in progress. The HIT panel was negative and PE was ruled out. She had a total of five sessions of plasmapheresis and mycophenolate was switched to cyclophosphamide/mesna. She developed acute upper gastrointestinal bleeding requiring three units of packed red cell transfusion and endoscopy did not reveal active bleeding or ulcers. Prednisone was tapered down to 25 mg daily orally and she was placed on intravenous pantoprazole.

Her blood pressure continued to rise to 160 mmHg/100 mmHg with flash pulmonary edema making extubation challenging and requiring re-intubation after successful extubation. She required several intubations and during her last re-intubation, a multi-disciplinary meeting was held, and a decision was made to extubate only after blood pressure is controlled and she was at her dry weight.

She became agitated, restless, and confused, with waxing and waning consciousness, and developed headaches and hallucinations. Concerns for steroid-induced psychosis vs. neuropsychiatric SLE were raised. She was tapered off prednisone and was continued on bi-weekly cyclophosphamide for induction therapy of lupus nephritis.

**Figure 2:**
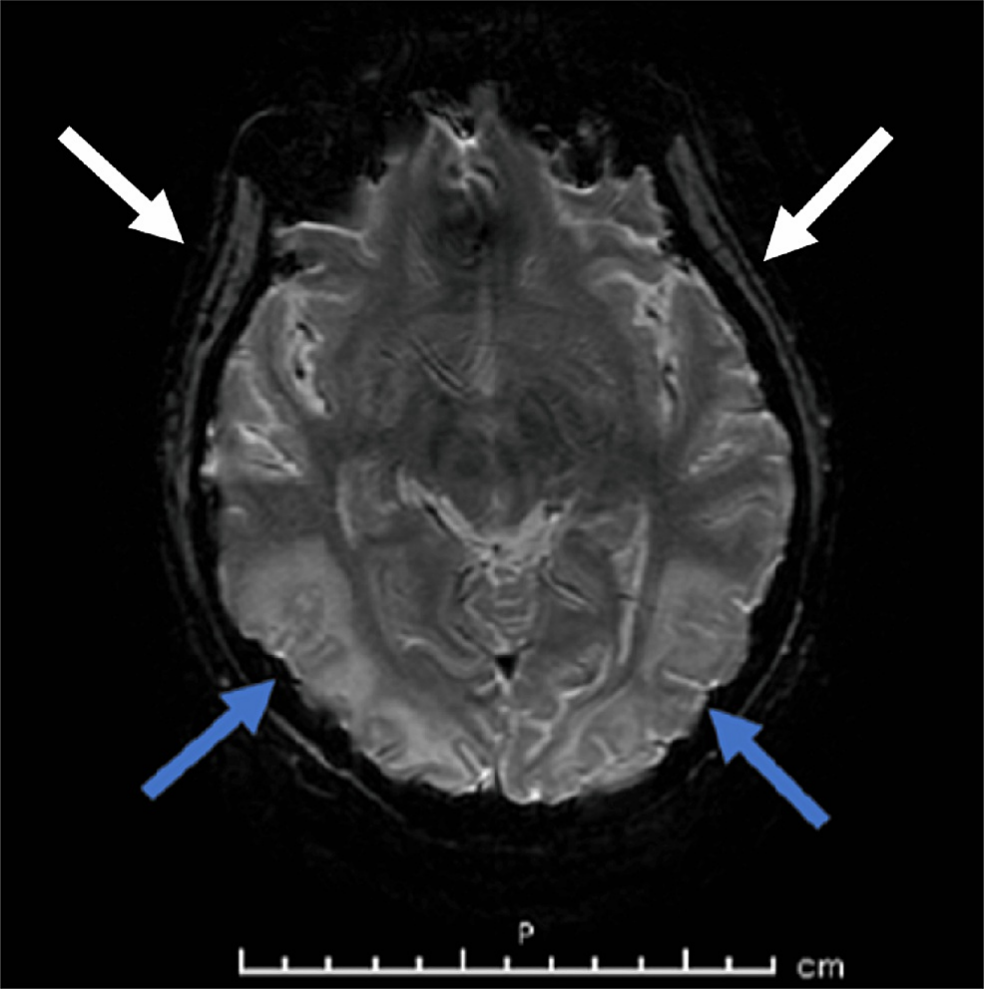
Non-contrast MRI of the brain with posterior (blue arrows) and frontoparietal (white arrows) extensive bilateral cerebral and cerebellar deep white matter high-intensity signals.

After her second dose of cyclophosphamide, her mentation continued to worsen and her blood pressure remained uncontrolled despite maximum doses of amlodipine, clonidine, hydralazine, isosorbide dinitrate, labetalol, and losartan. She was also placed on a nicardipine drip with a goal blood pressure of 120/80 mmHg. A non-contrast head CT scan showed extensive bilateral frontoparietal and occipital cortical and subcortical hypoattenuation foci suggestive of lupus cerebritis versus PRES and a non-contrast MRI showed extensive bilateral cerebral and cerebellar deep white matter high-intensity signals, as shown in Figure 2.

Cyclophosphamide was held and her mentation began to improve. She was successfully extubated and discharged to a rehabilitation center. Her mentation gradually improved over a period of two weeks. Her blood pressure remained only fairly controlled and she was continued on maximum doses of oral blood pressure medications and four times weekly dialysis. She was replaced on mycophenolate mofetil, low-dose prednisone, and hydroxychloroquine with control monthly follow-up and control of medication side effects as needed.

## Discussion

PRES was first described in the mid-1990s as a clinical syndrome with neurological signs and symptoms [1]. More recent studies have identified radiological findings associated with PRES. Different imaging patterns have been described with PRES, including holohemispheric watershed, superior frontal sulcus, dominant parietal/occipital, and partial and/or asymmetric PRES. It can affect the posterior and anterior aspects of the brain [2]. It is described as reversible but new reports of permanent neurological impairment and mortality have been reported [3]. It usually affects females more and usually middle-aged patients but the age range can vary from four years to 90 years [4].

The exact pathophysiological mechanism of PRES is not known. Some hypotheses described are infarcts in the brain from vasoconstriction, fluid accumulation in the brain from extravasation due to disruption in the blood-brain barrier, endothelial damage, and failure of autoregulation [4]. The cerebral white matter is susceptible to edema since it has loosely packed nerve fibers. Unlike the carotid vessels, the vertebrobasilar system is deficient in adrenergic innervation, making it susceptible to PRES [5]. Another hypothesis is an increase in total volume, which may occur in blood transfusions, with an increase in perfusion pressure and hence leading to edema. Severe anemia can also cause endothelial damage from inadequate oxygen supply to the brain [6]. Certain drugs, especially when used in combination, including cyclophosphamide, have been reported to cause endothelial damage or dysfunction [7].

Our patient had three units of packed red blood cell transfusion while she was fluid overloaded, which could lead to acute cerebral hyper-perfusion, but she was on daily hemodialysis and her mentation only worsened after she had a second dose of cyclophosphamide, which makes it a culprit in the development of PRES in this patient.

## Conclusions

Reports of PRES induced by cyclophosphamide are rare and reported once in a background of lupus nephritis with quiescent disease activity in a case report. PRES is not always confined to the posterior aspect of the brain. A combination of disease states and associated treatment regimens can lead to PRES. Prompt diagnosis and treatment with early identification of clinical presentation and imaging are vital because the management relies on discontinuing the triggers or managing the underlying diseases. Most patients will recover completely once the symptoms are controlled. If it is not treated promptly, permanent neurological deficits and death can occur.
